# MicroRNA-27a Is Induced by Leucine and Contributes to Leucine-Induced Proliferation Promotion in C2C12 Cells

**DOI:** 10.3390/ijms140714076

**Published:** 2013-07-08

**Authors:** Xiaoling Chen, Zhiqing Huang, Daiwen Chen, Ting Yang, Guangmang Liu

**Affiliations:** Key Laboratory for Animal Disease-Resistance Nutrition of China Ministry of Education, Institute of Animal Nutrition, Sichuan Agricultural University, Chengdu 611130, Sichuan, China; E-Mails: xlchen@sicau.edu.cn (X.C.); dwchendw@hotmail.com (D.C.); tingyangcn@163.com (T.Y.); lguangmang@163.com (G.L.)

**Keywords:** leucine, C2C12 cells, miR-27a, myoblast proliferation

## Abstract

Leucine, a branched chain amino acid, is well known to stimulate protein synthesis in skeletal muscle. However, the role of leucine in myoblast proliferation remains unclear. In this study, we found that leucine could promote proliferation of C2C12 cells. Moreover, expressions of miR-27a and myostatin (a *bona fide* target of miR-27a) were upregulated and downregulated, respectively, following leucine treatment. We also found that miR-27a loss-of-function by transfection of a miR-27a inhibitor suppressed the promotion of myoblast proliferation caused by leucine. Our results suggest that miR-27a is induced by leucine and contributes to leucine-induced proliferation promotion of myoblast.

## 1. Introduction

The development of skeletal muscle (myogenesis) is orchestrated by myoblast proliferation, withdrawal from the cell cycle, differentiation and fusion into multinuclear myotubes and then myofibers [1,2]. Therefore, myoblast proliferation is an early cellular event critical for skeletal muscle development. However, little is known about the impact of nutrients on this process. The branched chain amino acid leucine is an essential amino acid which cannot be synthesized by animals and humans. Although leucine is well known to stimulate muscle protein synthesis [3–6], its effect on myoblast proliferation remains unclear.

MicroRNAs (miRNAs) are highly conserved, short non-coding RNAs that regulate gene expression at the posttranscriptional level [7–10]. Growing evidence supports a role of miRNAs in various aspects of skeletal myogenesis [11]. The miR-27 family consists of miR-27a and miR-27b, which are transcribed from different chromosomes and differ in only one nucleotide at the 3′ end. Our recent study has shown that miR-27a can promote myoblast proliferation through targeting myostatin, a critical inhibitor of skeletal myogenesis [12]. In the present study, we demonstrated that miR-27a is induced by leucine and contributes to leucine-induced proliferation promotion in C2C12 cells.

## 2. Results

### 2.1. Effect of Leucine on Myoblast Proliferation

**Figure 1:**
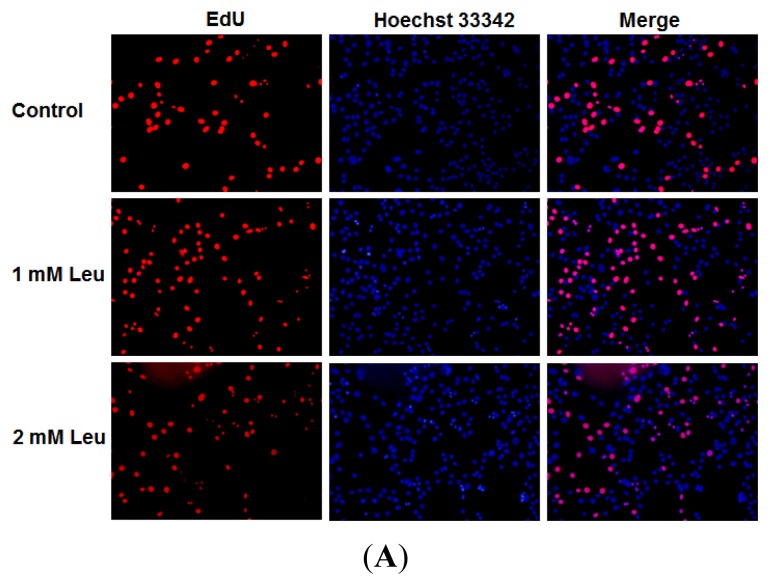

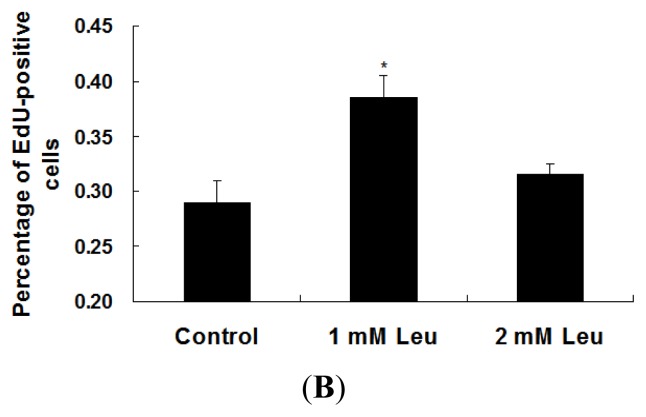
Effect of leucine on myoblast proliferation. C2C12 cells were seeded in a 24-well plate at a density of 1.0 × 10^4^ cells per well. After 48 h, the cells were starved in high-glucose DMEM for 4 h. The cells were subjected to treatment in the same starvation media by adding 0 mM (control), 1 mM or 2 mM of leucine. Cell proliferation was evaluated by EdU proliferation assay after 3.5 h of leucine treatment. (**A**) Proliferating C2C12 cells were labeled with EdU. The Click-it reaction revealed EdU staining (red). Cell nuclei were stained with Hoechst 33342 (blue). The images are representative of the data obtained; (**B**) The percentage of EdU-positive C2C12 cells were quantified. Results were presented as mean ± SE (*n* = 6). *p < 0.05.

The baseline concentration of leucine in DMEM (Invitrogen cat. No. 11995065) used in this study is 0.802 mM. To investigate the effect of leucine on myoblast proliferation, C2C12 cells were supplemented with different concentrations of leucine and EdU incorporation experiments were performed to assess its proliferation. As shown in Figure 1, the proliferation-promoting effect was obvious when supplementing with 1 mM leucine, compared with the unsupplemented control. In the following studies, leucine was supplemented with 1 mM.

### 2.2. Effect of Leucine on Expressions of miR-27a and Myostatin

**Figure 2:**
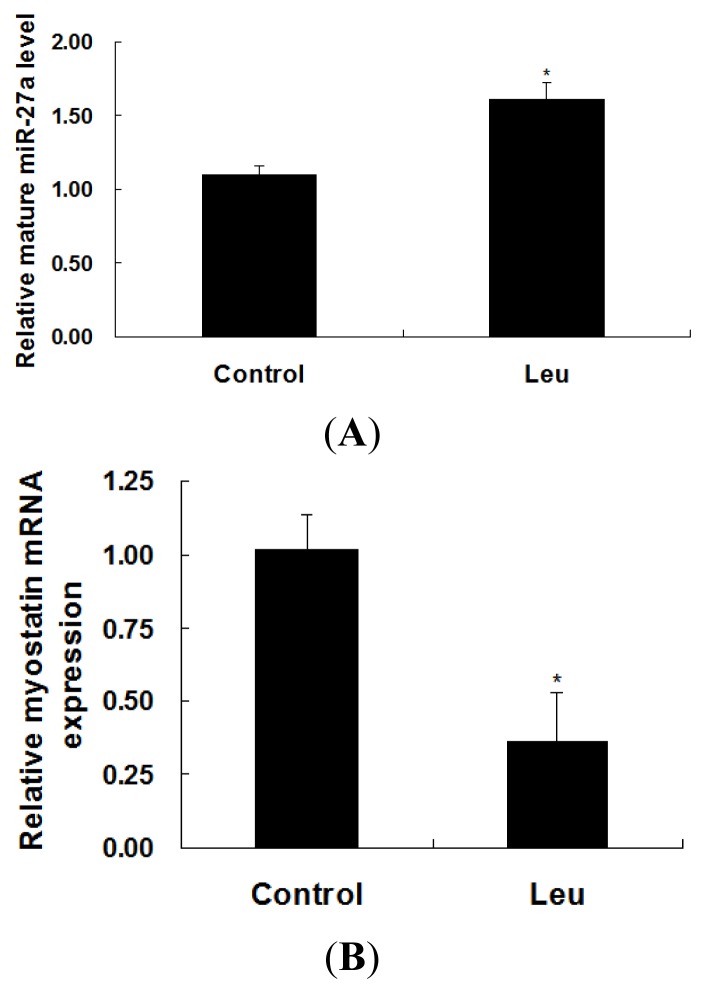
Effect of leucine on expressions of miR-27a and myostatin. C2C12 cells were seeded in a 24-well plate at 1.0 × 10^4^ cells per well. After 48 h, the cells were starved in high-glucose DMEM for 4 h and then supplemented with or without leucine (1 mM) for another 3.5 h in the same starvation media. Mature miR-27a level (**A**) and myostatin mRNA (**B**) were determined using real-time quantitative PCR. Samples were performed in duplicate. The amount of mature miR-27a and *myostatin* mRNA were normalized to the amount of U6 snRNA and *GAPDH* mRNA, respectively. The data were expressed as mean ± SE from three independent experiments. *p < 0.05.

Expressions of miR-27a and myostatin in leucine-supplemented C2C12 cells were determined by real-time quantitative PCR. As shown in Figure 2, levels of miR-27a and myostatin mRNA were increased and decreased, respectively, following leucine supplementation.

### 2.3. Promotion of Myoblast Proliferation by Leucine Is Reduced by miR-27a Inhibition

**Figure 3:**
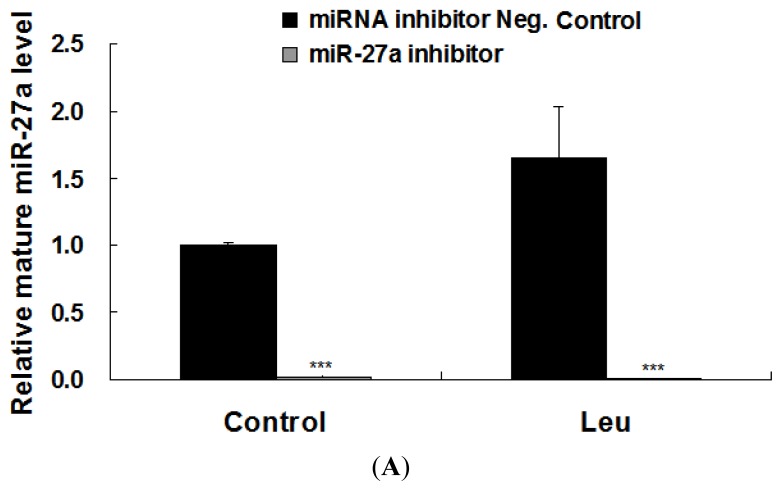

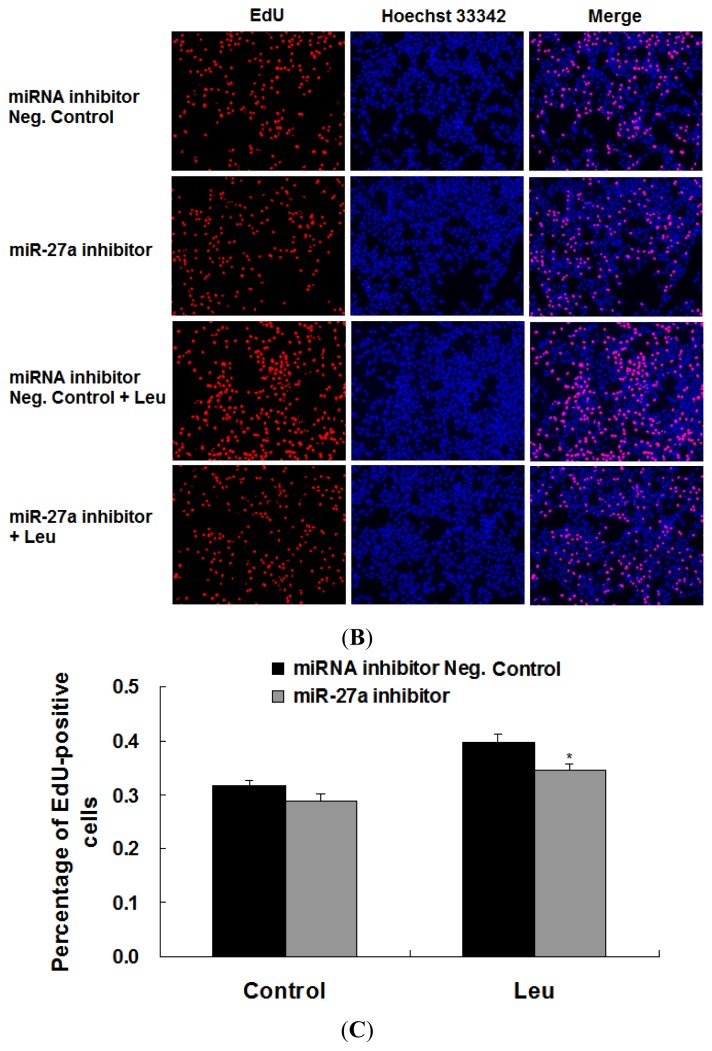
Promotion of myoblast proliferation by leucine is reduced by miR-27a inhibition. C2C12 cells were seeded in a 24-well plate at a density of 1.0 × 10^4^ cells per well. After 48 h, the cells were transfected with 100 nM of either miRNA inhibitor Negative Control or miR-27a inhibitor. Transfection mix was removed 6 h later and cells were grown in DMEM/10%FBS medium. Twenty-four hours after the transfection, the cells were starved in high-glucose DMEM for 4 h and then supplemented with or without leucine (1 mM) for another 3.5 h in the same starvation media. (**A**) The amount of mature miR-27a against U6 snRNA was measured by real-time quantitative PCR. Data were mean ± SE from three independent experiments performed in duplicate; (**B**) Proliferating C2C12 cells were labeled with EdU. The Click-it reaction revealed EdU staining (red). Cell nuclei were stained with Hoechst 33342 (blue). The images are representative of the data obtained; (**C**) The percentage of EdU-positive C2C12 cells were quantified. Results were expressed as mean ± SE (*n* = 6). *p < 0.05; ***p < 0.001.

Next, we examined whether the induction of miR-27a could be relevant for the proliferative effect of leucine on C2C12 myoblasts. To accomplish this aim, C2C12 cells were transfected an inhibitor of miR-27a followed by leucine supplementation. As shown in Figure 3A, mature miR-27a level in C2C12 cells transfected with miR-27a inhibitor was significantly lower than that in cells transfected with miRNA inhibitor Negative Control. As shown in Figure 3B, miR-27a inhibitor attenuated the promotion of myoblast proliferation caused by leucine. Quantitative analysis demonstrated that this change is statistically significant (Figure 3C).

## 3. Discussion

The biological function of leucine in the control muscle protein synthesis is well established [4]. More recently, Averous *et al.* demonstrated that leucine limitation inhibits the differentiation of both C2C12 myoblasts and primary satellite cells [13]. Here, we showed that leucine could promote proliferation of C2C12 myoblasts. These findings suggest that leucine plays more functional roles beyond the fundamental role as a substrate for muscle protein synthesis.

It has been well established that nutrients can regulate the expression of protein-coding genes [14]. However, growing evidence has accumulated supporting a role for nutrients in the regulation of miRNA expression [15–18]. In the present study, we observed that mature miR-27a was significantly elevated following leucine treatment in C2C12 cells. Myostatin is a member of the transforming growth factor-β (TGF-β) superfamily and is known as a critical inhibitor of skeletal myogenesis [19–22]. Recently, myostatin has been shown to be a *bona fide* target of miR-27a [12]. In this study, we showed that leucine treatment downregulated the transcriptional level of myostatin. Taken together, these studies indicate that nutrients regulate the expression not only of protein-coding genes but also of miRNAs.

Experimental data obtained by our group have provided evidence that miR-27a can promote myoblast proliferation [12]. In this study, we showed that leucine had a proliferation-promoting effect on C2C12 cells and miR-27a was induced by leucine. These two lines of evidence indicate that leucine-induced upregulation of miR-27a may contribute to leucine-induced proliferation promotion of myoblast. In this study, an approximately 9% reduction of EdU-positive cells was observed in miR-27a inhibitor-transfected C2C12 cells compared to the control, although the difference was not statistically significant (*p* = 0.15). We also showed that inhibition of the endogenous miR-27a repressed the proliferation promotion of C2C12 cells caused by leucine. These results indicated that miR-27a contributed to leucine-induced proliferation promotion of myoblast.

## 4. Materials and Methods

### 4.1. Reagents

L-leucine (Leu) was purchased from Sigma (St. Louis, MO, USA). TaqMan^®^ MicroRNA Assay kit specifically designed for miR-27a was from Applied Biosystems (Foster, CA, USA). Click-iT EdU Alexa Fluor 594 Imaging Kit was from Invitrogen (Carlsbad, CA, USA).

### 4.2. Cell Culture and Transfection

The mouse myoblast cell line C2C12 (CRL-1772) was obtained from the American Type Culture Collection (ATCC, Rockville, MD, USA). C2C12 cells were cultured in Dulbecco modified Eagle medium (DMEM) (Invitrogen, Carlsbad, CA, USA) supplemented with 10% fetal bovine serum (FBS) (Invitrogen, Carlsbad, CA, USA) at 37 °C in the 5% CO_2_, humidified atmosphere. C2C12 cells were transfected with miRNA inhibitor Negative Control or miR-27a inhibitor (RiboBio, Guangzhou, China) by using Lipofectamine 2000 (Invitrogen, Carlsbad, CA, USA) according to the protocols of the manufacturer.

### 4.3. Quantification of mRNA Expression

Total RNA was extracted from the adherent cultured C2C12 cells using TRIzol reagent (Invitrogen, Carlsbad, CA, USA) according to the instructions provided by the manufacturer. RNA quantity and quality were determined spectrophotometrically using a Beckman Coulter DU800 (Beckman Coulter, Fullerton, CA, USA). One microgram of total RNA from each sample was then converted into cDNA by using a PrimeScript^®^ RT reagent Kit with gDNA Eraser (TaKaRa, Dalian, China). Real-time quantitative PCR was carried out in a 7900HT real-time PCR system (384-cell standard block) (Applied Biosystems, Foster, CA, USA) using the iTaq SYBR Green Supermix with ROX (Bio-Rad, Hercules, CA, USA) in a final volume of 10 μL. The gene specific primers are listed in Table 1. Conditions for amplification were 2 min at 50 °C, 10 min at 95 °C, followed by 45 cycles of 15 s at 95 °C, and 30 s at 58 °C for primer annealing and elongation. A single sharp peak was observed in the melting curve and a single band of the expected size was observed in the agarose gel. Identities of the products were confirmed by DNA sequencing. Relative mRNA expression level was analyzed using the comparative Ct method [23], with *GAPDH* as the internal control for normalization.

### 4.4. Detection of miRNA Expression

Total RNA was isolated from cells with TRIzol reagent (Invitrogen). The concentration and quality of the isolated RNA were determined using a Beckman Coulter DU 800 Spectrophotometer (Beckman Coulter, Fullerton, CA, USA). cDNA synthesis was performed on 10 ng of total RNA with a TaqMan^®^ miRNA Reverse Transcription Kit (Applied Biosystems, Foster, CA, USA). Expression of mature miR-27a was assayed using TaqMan^®^ MicroRNA Assay kit (Applied Biosystems, Foster, CA, USA) specifically designed for miR-27a in a 7900HT real-time PCR system (384-cell standard block) (Applied Biosystems, Foster, CA, USA) following the manufactures’ instructions. The relative expression level of mature miR-27a was normalized to U6 snRNA (Applied Biosystems, Foster, CA, USA) by the comparative Ct method [23].

### 4.5. EdU Proliferation Assay

5-ethynyl-2′-deoxyuridine (EdU) is a nucleoside analog of thymidine whose incorporation can be used to label cells undergoing DNA replication. Proliferating C2C12 cells were evaluated by using the Click-iT EdU Alexa Fluor 594 Imaging Kit (Invitrogen, Carlsbad, CA, USA) according to the manufacturers’ instructions. Briefly, C2C12 cells were incubated with 10 μM EdU for 3 h at 37 °C, fixed with 3.7% formaldehyde for 15 min, and treated with 0.5% Triton X-100 for 20 min at room temperature. After washing twice with PBS containing 3% BSA, the cells were reacted with Click-iT reaction cocktail for 30 min. Subsequently, cell nuclei were stained with Hoechst 33342 (Invitrogen, Carlsbad, CA, USA) at a concentration of 5 μg/mL for 30 min. The images were acquired by fluorescence microscopy and overlapped using Image-Pro Plus (Version 6.0.0.260, Media Cybernetics, Inc., Tokyo, Japan).

### 4.6. Statistical Analysis

Data were expressed as mean ± SE. One-way ANOVA and Tukey’s tests (SPSS Inc., Chicago, IL, USA) were performed to assess the statistical significance between treatments. Statistical significance was defined at a level of *p* < 0.05.

## 5. Conclusions

In this study, we demonstrated that leucine could promote myoblast proliferation and induce the expression of miR-27a in C2C12 cells. The induction of miR-27a was also demonstrated to contribute to leucine-induced proliferation promotion of myoblast. The present study not only shed light on understanding the molecular mechanisms of leucine in myoblast proliferation, but also provided a useful reference for mechanism research of nutrients.

## Figures and Tables

**Table 1:** List of genes, primer sequences, GenBank accession numbers, and product sizes in this study.

Gene name	Primer	Sequence	GenBank ID	Product size
*Myostatin*	Forward	5′-GATGGGACTGGATTATCGC-3′	**NM_010834**	102 bp
	Reverse	5′-GCACAAGATGAGTATGCGG-3′		

*GAPDH*	Forward	5′-AGGGCATCTTGGGCTACAC-3′	**NM_008084**	211 bp
	Reverse	5′-TGGTCCAGGGTTTCTTACTCC-3′		
